# Highly Aligned Ni-Decorated GO–CNT Nanostructures in Epoxy with Enhanced Thermal and Electrical Properties

**DOI:** 10.3390/polym14132583

**Published:** 2022-06-25

**Authors:** Chenxi Hu, Hongnan Zhang, Nigel Neate, Michael Fay, Xianghui Hou, David Grant, Fang Xu

**Affiliations:** 1 Advanced Materials Research Group, Faculty of Engineering, University of Nottingham, Nottingham NG7 2RD, UK; cxhu@cauc.edu.cn (C.H.); ezxhz3@nottingham.ac.uk (H.Z.); nigel.neate@nottingham.ac.uk (N.N.); michael.fay@nottingham.ac.uk (M.F.); xianghui.hou@nottingham.ac.uk (X.H.); david.grant@nottingham.ac.uk (D.G.); 2 College of Science, Civil Aviation University of China, Tianjin 300300, China; 3 Nanoscale and Microscale Research Centre (nmRC), University of Nottingham, University Park, Nottingham NG7 2RD, UK

**Keywords:** Ni-decorated GO–CNT nanostructures, epoxy-based composites, aligned distribution of NiGNT nanostructures, anisotropic thermal and electrical performances

## Abstract

In this study, graphene oxide–carbon nanotubes nanostructures decorated with nickel nanoparticles (NiGNT) were prepared through the molecular-level-mixing method, followed by a reduction process, and then applied as reinforcements to enhance the epoxy resin matrix. The ferromagnetism of the Ni nanoparticles allowed NiGNT nanostructures to be vertically aligned within the composite with the assistance of a magnetic field. Due to the alignment distribution of the NiGNT, the composites demonstrated enhanced anisotropic thermal and electrical conduction performances, compared with pure epoxy and randomly distributed composites. The aligned distribution of NiGNT–epoxy composites displayed 2.7 times higher thermal conductivity and around 10^4^ times better electrical conduction performance, compared with pure epoxy. The thermal expansion of NiGNT–epoxy composite was also restricted in the aligned direction of NiGNT nanostructures. Thus, NiGNT–epoxy composites show great potential as future aerospace, aviation, and automobile materials.

## 1. Introduction

With the increasing requirements for lighter materials in aerospace, aviation, and automobile fields, polymer matrix composites (PMCs) have been widely investigated [1,2]. Epoxy resin, as one of the most widely used polymer materials, exhibits facile processability, as it is in a liquid state until cured and has good thermal stability as well as high modulus and strength after thermal curing [3]. However, traditional epoxy-based products with poor electrical and thermal conductivity are not suitable for many potential applications without further improvement. For example, when lighting hits a polymer matrix structure applied as aerospace or aviation materials, the structure would burn through if the electric current could not be effectively transferred [4]. Thus, different strategies have been introduced to improve the thermal and electrical conduction performances of PMCs, and adding fillers has been regarded as a good choice [5,6,7].

Among the filler materials, carbon materials, i.e., carbon fibres (CFs) [8,9,10], graphene [5,11,12], and carbon nanotubes (CNTs) [13,14] have been regarded as promising candidates for reinforcing the epoxy resin matrices due to their unique properties (high theoretical electrical and thermal performances as well as high modulus and strength [15,16]). Compared with carbon fibres, carbon nanomaterials (one-dimensional CNTs and two-dimensional graphene materials) have shown greater potential as reinforcements in polymer matrix due to larger surface area and better interface with the matrix [17]. Until now, the reported results of the obtained carbon material enhanced epoxy composites indeed exhibited improved performances but still lower than the theoretical predictions. The possible reasons are that (i) the dispersion of the carbon material fillers is not as uniform as predicted in the matrix, especially the π–π interactions between CNT walls and graphene which would lead to agglomeration during the dispersion, and (ii) there is a poor interface between fillers and matrices in some cases [18].

Several strategies have been explored to improve the performances of carbon-nanomaterial-reinforced matrices. The incorporation of functionalised CNTs into epoxy has shown enhanced thermal and electrical conductivity [19,20,21], and this could be ascribed to the functional groups (such as –OH and –COOH) with negative charges, resulting in better dispersion of CNTs by overcoming the π–π interactions through electrostatic repulsion. Magnetic-field-assisted alignment of fillers is also an efficient approach to preparing the anisotropic distribution of fillers, especially in a certain direction in the matrices (such as the thickness direction). Wang et al. reported alignment of graphene [5,11] and carbon fibres [22] in the matrix with the assistance of a magnetic field, and the obtained composites displayed enhanced electrical and mechanical properties. Tannenbaum et al. demonstrated the anisotropic conductivity of epoxy composite reinforced by aligned magnetic CNTs [14]. As pristine carbon materials are not ferromagnetic and cannot be orientated by a magnetic field, decoration with ferromagnetic particles (i.e., Ni, Fe_3_O_4_, and other ferrites) has become a common method [23,24,25] to produce magnetised carbon materials. However, traditional decoration methods are time-consuming and complicated, which restricts their further applications [23,26].

Herein, a freeze-drying-based preparation method combining the molecular-level-mixing (MLM) and a subsequent reduction process was developed to prepare GO–CNT nanostructures uniformly decorated with Ni nanoparticles (NiGNTs). The obtained nanostructures were then used as fillers in an epoxy resin matrix with and without the assistance of a weak magnetic field. Due to the ferromagnetism of Ni nanoparticles decorated on the GNT nanostructures, the NiGNT samples could be vertically aligned within the composite with the assistance of this magnetic field. Accordingly, the performances of as-prepared composites could be enhanced in the designed direction. A comprehensive characterisation was carried out with the obtained composites, and the relevant thermal and electrical properties were also carefully investigated.

## 2. Materials and Methods

### 2.1. Materials

Graphene oxide (GO) dispersions were purchased from William Blythe Limited (Accrington, UK), carbon nanotubes (CNTs) were purchased from Carbon Nanotubes Plus (Madisonville, KY, USA), and Table 1 displays the physicochemical characteristics of GO and CNTs. Nickel acetate [Ni(CH_3_COO)_2_], sulfuric acid (H_2_SO_4_, 98%) and acetone were purchased from Sigma-Aldrich (Sigma-Aldrich, Gillingham, UK), while nitric acid (HNO_3_) was purchased from Fisher Scientific. Epoxy resin was supplied by Aktinium Technologies Limited and was divided into part A and part B (part B was regarded as the hardener), Table 2 shows the properties of each parts. Liquid N_2_ (LN_2_), pure Ar gas, and Ar/H_2_ mixed gas (5% H_2_ + 95% Ar) were supplied by BOC Gas Cylinders.

### 2.2. Preparation of Ni-Decorated GO–CNT (NiGNT) Nanostructures

#### 2.2.1. Acid Treatment of CNTs

CNTs from Carbon Nanotubes Plus were firstly acid-treated via a simple method [27]. Specifically, a flask containing 0.5 g CNTs, 10 mL HNO_3_, and 5 mL H_2_SO_4_ was kept at 130 °C in an oil bath with a reflux system for 25 min. Afterwards, the acid-treated CNTs were filtrated, rinsed with distilled water, and dried in a vacuum oven overnight.

#### 2.2.2. Synthesis of NiGNT Nanostructures

**Figure 1:**
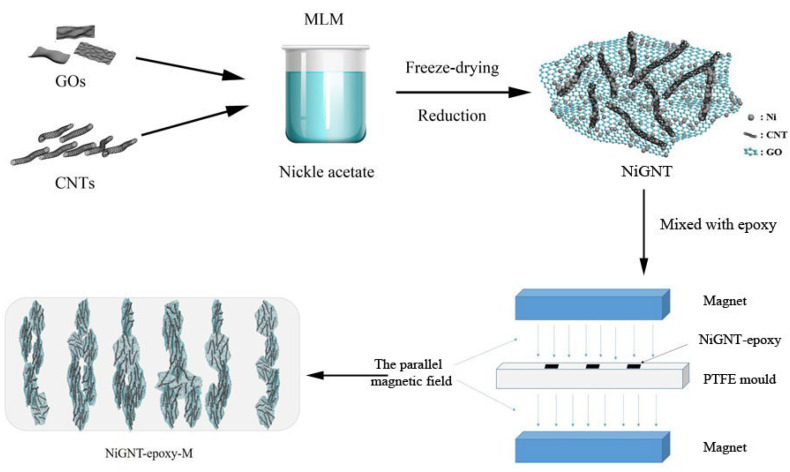
Schematic of the preparation of NiGNT nanostructures and the alignment of NiGNT–epoxy composites.

As shown in Figure 1, Ni-decorated GNT (NiGNT) nanostructures were obtained through the MLM method with subsequent freeze-drying and reduction processes [27]. Ni(CH_3_COO)_2_ was dissolved in 50 mL H_2_O through magnetic stirring, while acid-treated CNTs and GOs were mixed with a ratio of 1:1 and dispersed under ultrasonication for half an hour to obtain a well-dispersed mixture. The obtained carbon mixture was then added to the nickel solution to obtain a weight ratio of Ni:C of 9:1 and stirred for another 2 h. After stirring, the suspension was quickly frozen under liquid nitrogen (LN_2_) atmosphere and was then freeze-dried via a Labconco FreeZone 76705 freeze dry system at −55 °C and 0.024 mBar. The obtained precursor powders were then reduced under H_2_/Ar atmosphere at 450 °C for 2 h in a tube furnace. The pure GO–CNT (GNT) sample was prepared in the same way as the NiGNT sample without the addition of Ni(CH_3_COO)_2_.

### 2.3. Preparation of Aligned NiGNT–Epoxy and Its Reference Composites

To prepare the epoxy-based composites, NiGNT nanostructures were uniformly mixed with epoxy part A and then cured after adding epoxy part B, as shown in Figure 1. Specifically, NiGNT nanostructures and epoxy part A were separately dispersed in acetone, and then poured together and mechanically stirred for 1 h. The well-mixed solution was then stirred and heated at 90 °C to completely remove acetone. After that, the epoxy part B was added to the mixture, and the resulting weight percentage of NiGNT within the composite ranged from 10 to 30 wt.%. The mixture was then poured into the PTFE mould. For the aligned NiGNT–epoxy composites, the mould was first placed in a parallel magnetic field with a magnetic strength of 50 mT for one hour and then transferred to an oven at 60 °C for one hour, followed by 90 °C for one hour, also in a magnetic field throughout the curing process. For the reference sample, the curing process was similar to the aligned composites just without the assistance of the magnetic field. As-prepared aligned NiGNT–epoxy composites are named M-NiGNT-X (where X refers to the weight percentage of NiGNT nanostructures in the epoxy matrix, i.e., M-NiGNT-10 was the 10 wt.% of aligned NiGNT in epoxy), while the reference composite with the same amount of NiGNT is named as NiGNT-X.

### 2.4. Characterisation

X-ray diffraction (XRD) results were obtained through the Bruker D8 Advance system (operated at 40 kV and 35 mA) using Cu K-α radiation as the X-ray source, between 5 and 80° 2θ range at a scanning step of 0.01°, and the time per step is 0.12 s. The morphologies of obtained powders and the cryogenic fracture surfaces of epoxy-based composites which were subsequently coated with 10 nm carbon using a sputtering device were recorded through a JEOL 7100 field-emission gun scanning electron microscope (SEM) with the working voltage of 15 kV. A JEOL 2100+ transmission electron microscope (TEM) equipped with a Gatan Ultrascan 1000XP CCD camera was used at a working voltage of 200 kV to acquire TEM images of NiGNT nanostructures, and images of epoxy-based composites at 80 kV. The TEM sample of NiGNT nanostructures was prepared by dispersing the as-prepared powders in ethanol under sonication and then dropping them onto holey carbon TEM grids, while all composite samples were cut using an ultramicrotome with a fine diamond blade into thin films of ca. 100 nm for observation. X-ray photoelectron spectroscopy (XPS) was carried out on a VG ESCALab Mark II spectrometer machine using Al K-α X-ray as an excitation source, with a wavelength of 1486.6 eV. It was performed under a condition of anode voltage of 12 kV and an emission current of 20 mA. A thermogravimetric (TGA) experiment was performed using TA Q600 SDT. The NiGNT sample was measured from 50 to 700 °C in air, while the epoxy-based composites were measured from 50 °C to 800 °C in air, and both with a ramp rate of 10 °C min^−1^. Thermal–mechanical analysis (TMA) was adopted to analyse the coefficient of thermal expansion (CTE) of the epoxy composites with TMA Q400 from room temperature to 100 °C with 5 °C min^−1^ under a N_2_ atmosphere. The thermal diffusivity (α) of all the epoxy-based composites was measured using a NETZSCH Light Flash Apparatus (LFA) 467 Hyper Flash instrument at room temperature. The density (*ρ*) of the as-prepared pellet was obtained through Archimedes’ principle. The specific heat capacities (*C_p_*) of all epoxy-based pellets were recorded with TA DSC2500. The thermal conductivity (K) of these samples was then calculated by the following equation: $\;K=\alpha \times \rho \times C_{p}$. EIS tests were measured by ModuLab XM Materials Test System (Solartron, Hampshire, UK). The EIS curves were measured at room temperature over the frequency range of 0.01 to 1 MHz. Impedance plots were analysed using the Zview software (Scribner Associates, Inc., Southern Pines, NC, USA). The values of resistivity (R) can be obtained from the intercept of the corresponding semicircle on the real part of the impedance. All of the thermal and electrical performance tests were measured three times for each sample.

## 3. Results and Discussion

### 3.1. Structure and Morphology Characterisation of NiGNT Nanostructures

**Figure 2:**
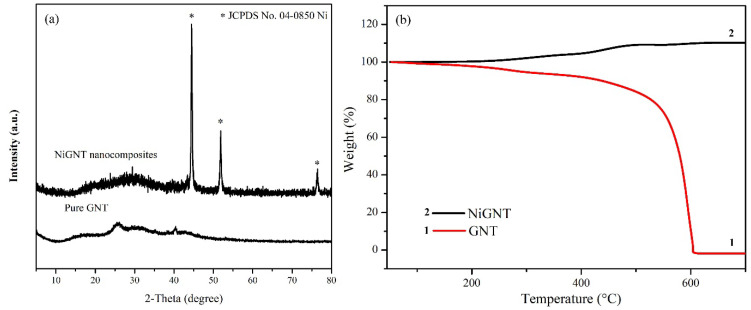
XRD patterns (**a**) and TGA results (**b**) of NiGNT and pure GNT samples.

Figure 2a displays XRD patterns of obtained NiGNT and pure GNT nanostructures. In the patterns of pure GNT, a broad peak ranging from 30 to 40 degrees could be detected, which was ascribed to the carbon content in the sample [28]. Meanwhile, three strong diffraction peaks at 44.5°, 51.8°, and 76.4° 2θ were observed in XRD patterns of NiGNT nanostructures, which indicates the (111), (200), and (220) planes of standard metallic Ni (ICDD PDF No. 00-04-0850). In addition, the broad peak around 30–40 degrees further confirmed the existence of carbon materials in the nanostructures. The XRD result confirmed that nickel acetate was completely reduced to Ni and the GNT remained under the treatment at 450 °C for 2 h in Ar/H_2_ atmosphere. TGA was introduced to investigate the composition of the as-prepared samples [29], and the results are displayed in Figure 2b. There was almost no mass remaining after 600 °C, as shown in curve 1 (pure GNT sample), which was ascribed to the full oxidation of carbon content. For the NiGNT sample (curve 2), a slight weight increase (around 13 wt.%) was found after the temperature reached 600 °C, and the weight change remained stable after that. This mass increase can be attributed to the oxidisation of Ni to the NiO phase in air. By considering the burning of carbon content in the sample, the calculated weight percentage of carbon content was 11.44% which was consistent with the designed proportion (Ni:C as 9:1).

**Figure 3:**
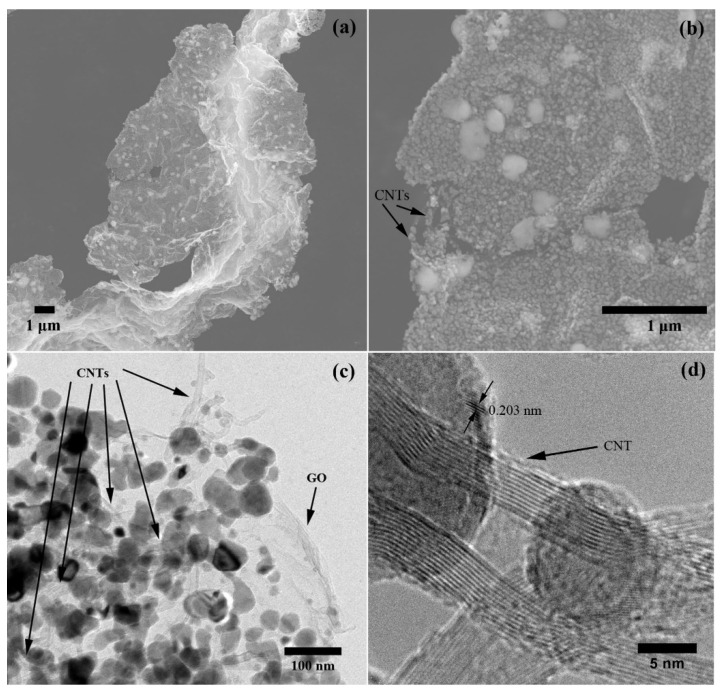
SEM (**a**,**b**) and TEM images (**c**,**d**) of NiGNT nanostructures.

The morphology of the NiGNT nanostructures was investigated using SEM and TEM techniques, and illustrative images are shown in Figure 3. In Figure 3a, well-dispersed graphene-layer-like structures can be clearly observed, indicating that there was no obvious agglomeration during the preparation process. Meanwhile, Ni nanoparticles were found uniformly coated on the grapheme-layer structures. Under higher magnification (Figure 3b), CNTs were detected on the edge of the nanostructure with Ni nanoparticles coated on them. From SEM images, the size of Ni nanoparticles which were uniformly coated on both GO and CNTs ranges from 20 to 50 nm, and there were still several large Ni particles around 100 nm. To further investigate the morphology and structure of the NiGNT samples, TEM images are displayed in Figure 3c,d. In the TEM image (Figure 3c), the GO layer structure can be easily found due to different contrast with the background, while the Ni nanoparticles can be detected as the dark features due to their high molar weight. In addition, CNTs were observed on the edge of the nanostructure, as well as between Ni nanoparticles and the GO layer. In the HRTEM image (Figure 3d), the multiwall structure of CNTs is clearly observed, and the number of walls was around 10–11. The Ni nanoparticles were attached to the CNTs, and the obtained typical lattice fringe spacing was measured to be 0.203 nm which could be ascribed to the (111) crystal plane of Ni (ICDD PDF No. 00-04-0850), and this was also consistent with the XRD result above.

**Figure 4:**
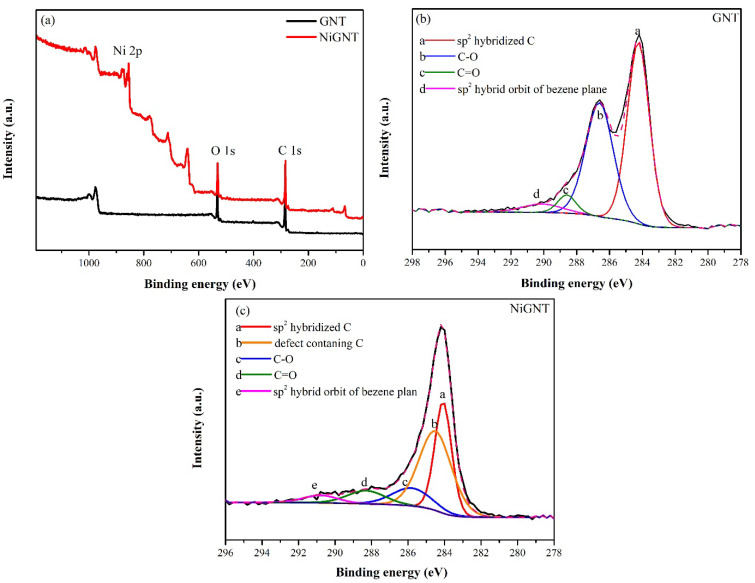
The XPS survey scan (**a**), and the deconvoluted C 1 s spectrum of GNT (**b**) and NiGNT (**c**) samples.

To further investigate the chemical composition of GNT and NiGNT nanostructures, XPS was introduced, and the results of both samples are shown in Figure 4. Survey scans of GNT and NiGNT samples are displayed in Figure 4a, and both spectra illustrate the characteristic peaks of C 1 s and O 1 s. Ni 2p characteristic peaks were found in the survey scan of the NiGNT sample which further confirmed the existence of the Ni element. The deconvoluted C 1 s spectra of both samples are shown in Figure 4b,c. In the deconvoluted C 1 s spectrum of the GNT sample (Figure 4b), the peak could be deconvoluted into the sp^2^-hybridised C located at 284.6 eV, the C−O (ether bonds) which corresponded to the hydroxyl groups at 286.3 eV, the C=O groups (ketone bonds) at 288.9 eV, and the sp^2^ hybrid orbit of benzene plane at 290.8 eV [30]. The existence of such O-containing groups could not simply prevent the agglomeration of graphene layers in solutions due to the electrostatic repulsion but provide suitable sites for metal ions nucleation as well. However, different deconvoluted peaks were observed in the deconvoluted C 1 s spectrum of the NiGNT sample (Figure 4c). The main C peaks in the NiGNT sample became the sp^2^-hybridised C (at around 284.0 eV) and defect-containing sp^2^-hybridised C (at around 285.0 eV), indicating that more C defects were obtained as a result of the reduction process in the preparation procedures [31]. Meanwhile, the oxygen-contained component—including the C−O (ether bonds) groups corresponded to the hydroxyl groups at 286.3 eV, the C=O groups (ketone bonds) at 288.9 eV, and the sp^2^ hybrid orbit of the benzene plane at 290.8 eV—could still be observed, but the intensity of such functional groups (especially the C−O groups) decreased dramatically in the NiGNT sample. This indicates that the C−O groups, in particular, were reduced during the reduction process.

### 3.2. Structure and Morphology Characterisation of NiGNT–Epoxy Composites

**Figure 5:**
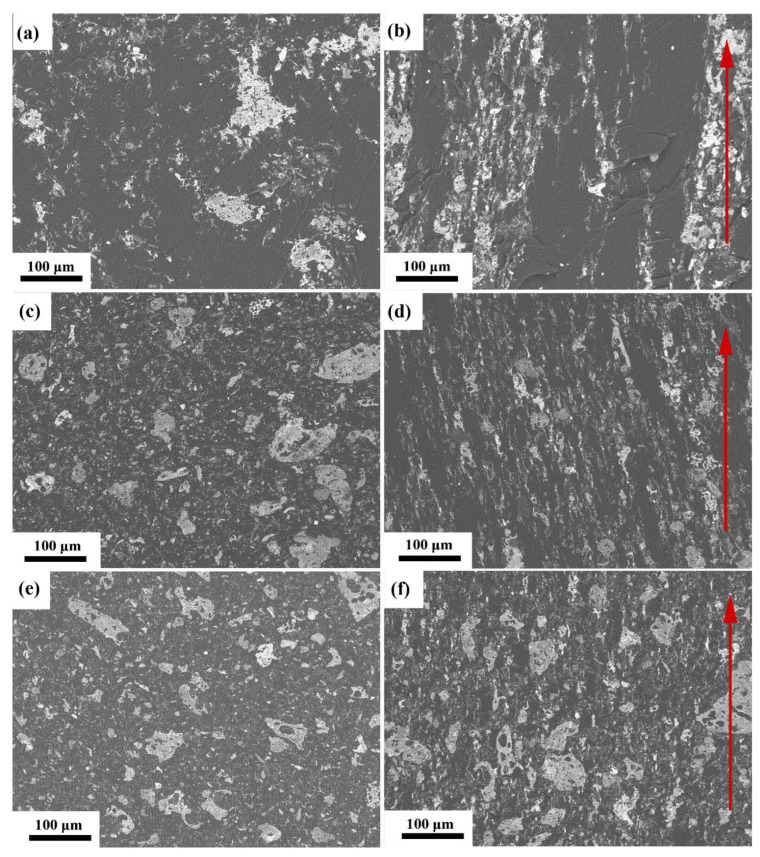
Back-scattering SEM images of the NiGNT-10 (**a**), M-NiGNT-10 (**b**), NiGNT-20 (**c**), M-NiGNT-20 (**d**), NiGNT-30 (**e**), and M-NiGNT-30 (**f**) composites; the red arrows indicate the direction of the magnetic field.

The distribution of the NiGNT nanostructures in the epoxy matrix, with and without magnetic field assistance, was analysed using SEM. The cross-sectional back-scattered electron images of all NiGNT–epoxy composites are shown in Figure 5, the red arrows indicate the directions of the magnetic field. As evident in the back-scattering images of 10-NiGNT–epoxy composites (Figure 5a,b), NiGNT nanostructures were randomly distributed among the epoxy matrix on the cross-section of the reference sample (Figure 5a), while their distribution was aligned parallel to the magnetic field direction in the M-NiGNT-10 sample (Figure 5b). The alignment distribution was parallel to the magnetic field direction. Meanwhile, both M-NiGNT-10 and its reference composites showed large spaces without NiGNT fillers which indicated that 10 wt.% of NiGNT fillers could not fully fill the epoxy matrix. Showing the increasing amount of filler ratio, Figure 5d,c illustrate the back-scattering SEM images of M-NiGNT-20, and its reference sample, respectively. In NiGNT-20 composites (Figure 5c), the NiGNT fillers were still randomly dispersed in the epoxy matrix. With the assistance of a magnetic field (Figure 5d), NiGNT fillers demonstrated uniform and aligned distribution which was similar to the 10 wt.% composites, but fewer empty spaces could be detected. In 30 wt.% NiGNT–epoxy composites (Figure 5e,f), a similar distribution trend of fillers was observed in both reference and M-NiGNT-30 composites but with fewer spaces, without NiGNT fillers. Thus, NiGNT nanostructures were uniformly distributed in an epoxy matrix with the help of an external magnetic field, and it was found that NiGNT nanostructures generated alignment 3D pathways in the matrix which should be beneficial to the electrical and thermal conduction.

**Figure 6:**
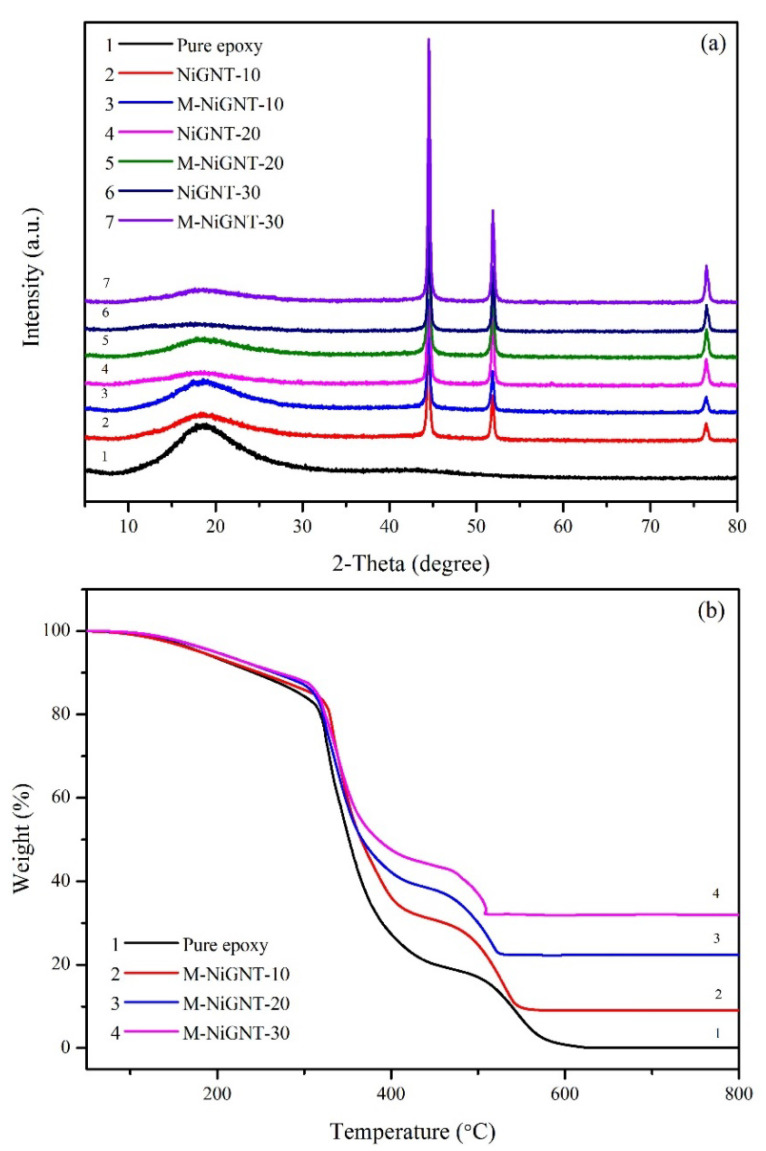
XRD patterns (**a**) and TGA (**b**) curves of the epoxy-based composites.

XRD results of the NiGNT-reinforced epoxy composites and their reference samples are shown in Figure 6a. A wide and broad peak located at around 10–30° was observed for the pure epoxy sample, which also appeared in all other NiGNT-reinforced epoxy composites. Meanwhile, the characteristic peaks of (111), (200), and (220) planes of Ni (ICDD PDF No. 00-04-0850) phase at 44.5°, 51.8°, and 76.4° 2θ were also detected in all NiGNT-reinforced composites. It is interesting to note that the intensities of epoxy were markedly different between the magnetic-field-assisted samples (3, 5, and 7) and the reference samples (2, 4, and 6). The intensities of all three reference samples were lower than the intensities of their corresponding magnetic-field-assisted samples (such as curves 2 compared with 3, curves 4 compared with 5, and curves 6 compared with 7). One possible reason for this phenomenon is that the alignment distribution of NiGNT nanostructures was obtained in the matrix (Figure 5), and this vertical distribution made fewer Ni nanoparticles facing upwards than the random distribution sample. Thus, a stronger intensity of Ni peaks in the reference sample was obtained, while less intensity of epoxy component could be observed. To further investigate the constitution of NiGNT–epoxy composite samples, TGA results are displayed in Figure 6b. Although the distribution of NiGNT nanostructures fillers was different among epoxy matrices with and without magnetic field, the weight ratio of NiGNT fillers was the same in each ratio group of composites. Therefore, only magnetic-field-assisted composites are demonstrated. As can be seen in curve 1 (Figure 6b), the pure epoxy sample displayed a continuous trend of weight loss until reaching zero which means all the samples were oxidised in the air after 600 °C. There were three weight decrease stages of pure epoxy samples in the whole TGA process. The first stage was located around 180–350 °C which indicates the first thermally oxidative degradation stage of the neat epoxy [32]. The other two sharp decreases at around 380 °C and 500 °C were attributed to further oxidation of macromolecular chains and the release of other small molecular degradation products [32,33,34]. For the other three NiGNT contained composites, TGA curves displayed similar trends as pure epoxy but with mass remaining which was ascribed to the NiO oxidised from Ni nanoparticles. The remaining mass of the M-NiGNT-10 composites was around 12 wt.% which was calculated as 9.4 wt.% of pure Ni, while this value for M-NiGNT-20 and M-NiGNT-30 composites was 18.5 wt.% and 27.8 wt.%, respectively. This was consistent with the initial design of the composite preparation stage.

**Figure 7:**
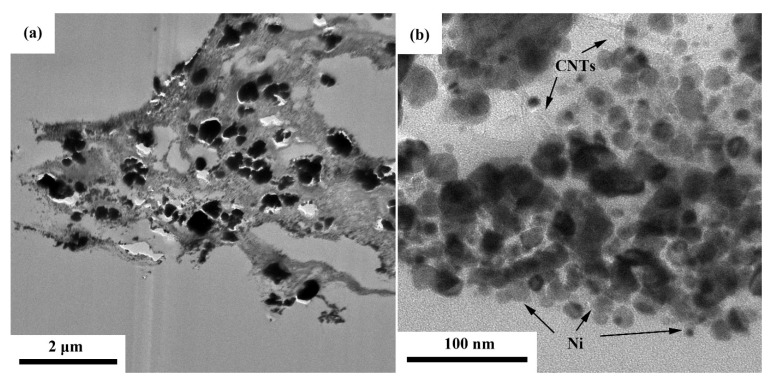
TEM image of NiGNT–epoxy composites under low (**a**) and high (**b**) magnification.

To further explore the distribution of NiGNT nanostructures in the epoxy matrix, TEM images of the M-NiGNT-10 sample cut using an ultramicrotome are displayed in Figure 7. As shown in Figure 7a, NiGNT nanostructures were clearly observed embedded in the epoxy matrix, and no obvious damage to the NiGNT was observed, indicating the integrity of the NiGNT nanostructure during the composite preparation process. The graphene-layer-like structure of NiGNT nanostructures was kept intact during stirring and solvent evaporation, and no void was observed around the interface of NiGNT and epoxy. In the high-resolution image (Figure 7b), the hollow tube structure of CNTs and dense particle structure of Ni were clearly observed, again, with no obvious void detected between epoxy and NiGNT nanostructures, suggesting a good interface between the NiGNT nanostructures and epoxy matrix. Few bright parts were observed mainly beside the large Ni particles; these were ascribed to some voids which resulted from the TEM sample preparation (attaching to or peeling off by the cutting parts).

### 3.3. Electrical Conductivity

**Figure 8:**
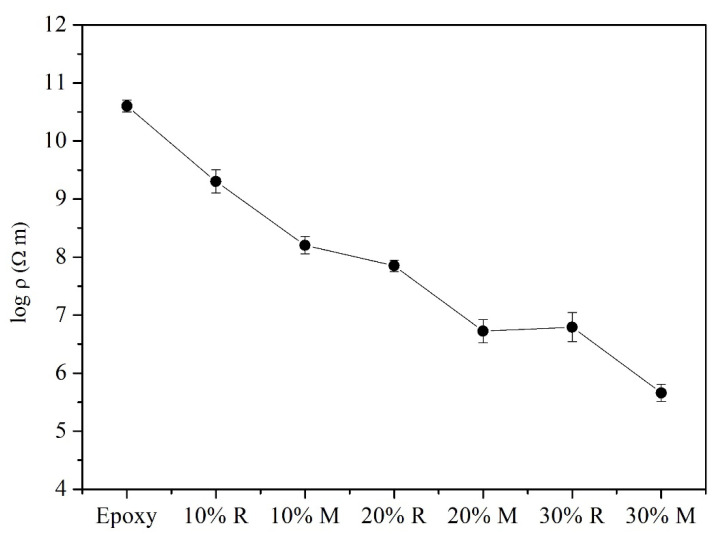
Electrical resistivity of pure epoxy and the NiGNT–epoxy composites with different ratios.

To investigate the electrical conduction property of the composite samples, the electrical resistivities were obtained through ModuLab XM Materials Test System, and the results are shown in Figure 8. For the pure epoxy sample, the obtained electrical resistance was over 10^10^ Ω m, which indicated the insulation instinct of the pure epoxy sample. After the addition of NiGNT nanostructures, a decreasing trend of electrical resistivity could be observed. Firstly, a minor decrease was found in the NiGNT-10 sample, around 2 × 10^9^ Ω m, and it further decreased to around 1.6 × 10^8^ Ω m in the M-NiGNT-10 sample. With the growth of the NiGNT filler amount, NiGNT-20 composites showed lower electrical resistivity. The electrical resistivity of the NiGNT-30 composite continued to decrease, and the value for M-NiGNT-30 sample reached 4.6 × 10^5^ Ω m. When compared with previous studies, the as-prepared M-NiGNT-30 composite displayed better electrical conduction performance, such as the aligned functionalised CNTs in epoxy via an electric field (~10^6^ Ω m) [14], the aligned multilayer graphene flakes in epoxy with an external electric field (~10^6^ Ω m) [11], and aligned Fe_3_O_4_-coated graphene nanoplatelets in epoxy via a weak magnetic field (~10^6^ Ω m) [5]. Therefore, it was concluded that the introduction of NiGNT nanostructures as reinforcements in the epoxy matrix could obviously increase the electrical conduction performance, especially in the aligned samples (M-NiGNT-30 sample), which was almost 10^5^ times higher than the pure epoxy sample.

### 3.4. Thermal Conductivity and the Coefficient of Thermal Expansion

**Figure 9:**
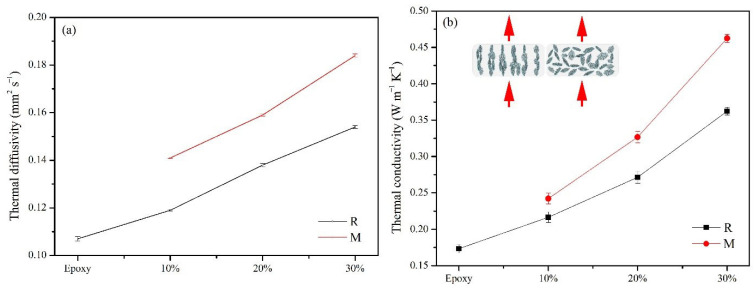
Thermal diffusivity (**a**) and thermal conductivity (**b**) of pure epoxy and the NiGNT–epoxy composites with different ratios.

To investigate the thermal properties of the as-prepared NiGNT–epoxy composites, thermal conductivity and CTE measurements were carried out, and the relevant results are displayed in Figure 9. From Figure 9a, it can be derived that the thermal diffusivities increased with the addition of NiGNT fillers, and the magnetic-field-assisted samples showed better thermal diffusivities when compared with each reference sample. Specifically, the pure epoxy resin displayed a thermal diffusivity of 0.107 mm^2^ s^−1^, and this value increased to 0.119 mm^2^ s^−1^ in the NiGNT-10 composites, which indicated that the addition of NiGNT nanostructures was beneficial to thermal conduction. Meanwhile, the M-NiGNT-10 composites showed further improvement in thermal diffusivity (0.141 mm^2^ s^−1^) after the magnetic field assistance in the preparation method. When the ratio of NiGNT nanostructures grew, the thermal diffusivities of NiGNT-20 and NiGNT-30 composites were 0.138 and 0.154 mm^2^ s^−1^, respectively, while these values for M- NiGNT-20 and M- NiGNT-30 composites were 0.159 and 0.184 mm^2^ s^−1^. This indicated the enhancement of NiGNT nanostructures in the epoxy matrix, as well as the further reinforcement of fillers’ alignment with the assistance of the magnetic field. The obtained thermal conductivities of all the epoxy-based composites are shown in Figure 9b. The pure epoxy sample displayed a thermal conductivity of around 0.173 W m^−1^ K^−1^ which is close to most reported values [35]. After the addition of NiGNT nanostructures, the thermal conductivity showed a similar increasing trend as thermal diffusivity, and it increased to 0.217 W m^−1^ K^−1^ for the NiGNT-10 sample and 0.242 W m^−1^ K^−1^ for the M-NiGNT-10 sample. Meanwhile, this increasing trend continued with an increase in filler ratios. When the weight ratio became 20 wt.%, the NiGNT-20 sample showed a thermal conductivity value of 0.271 W m^−1^ K^−1^, whereas this value was 0.326 W m^−1^ K^−1^ for the M-NiGNT-20 sample. With the increase in filler amounts, the obtained thermal conductivities demonstrated further increase, with thermal conductivity reaching 0.362 W m^−1^ K^−1^ for NiGNT-30 and 0.462 W m^−1^ K^−1^ for M-NiGNT-30, respectively. The obtained thermal conductivity of the M-NiGNT-30 composite was around 2.67 times higher than that of pure epoxy. It was found that the thermal conduction performance was effectively enhanced with the addition of NiGNT samples. Meanwhile, the alignment distribution of the NiGNT fillers which was confirmed by the SEM results (Figure 6) also demonstrated further improvement in thermal conductivity.

**Figure 10:**
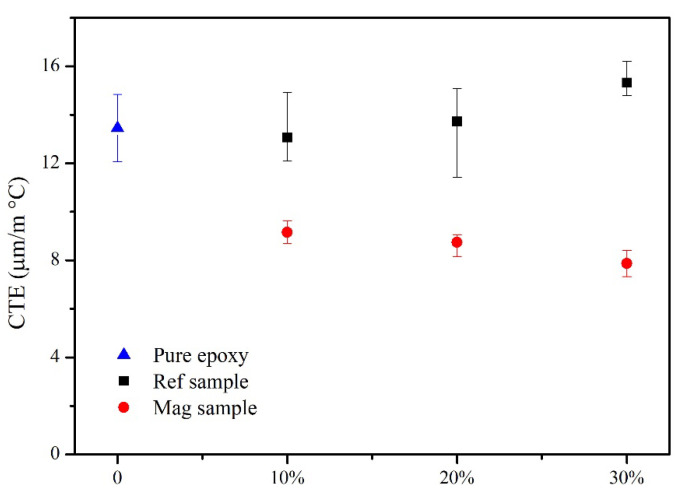
CTE of pure epoxy and the NiGNT–epoxy composites with different ratios.

The thermal expansion of NiGNT–epoxy composites alongside Z direction was measured under glass transition temperature (T_g_), and the CTEs of each sample are displayed in Figure 10. It was found that the pure epoxy sample (blue) had a CTE of around 13.45 µm m^−1^ °C^−1^, and the NiGNT–epoxy reference samples demonstrated a similar CTE when compared with the pure epoxy sample. This indicated that the random distribution of the NiGNT nanostructures did not obviously change the CTE of the epoxy matrix, nor did the increasing amount of NiGNT nanostructures effectively decrease the CTE. Furthermore, the M-NiGNT composites displayed additional enhancement in addition to that from increasing the filler ratios. The obtained CTE of the M-NiGNT-10 composites was 9.15 µm m^−1^ °C^−1^ which was lower than the NiGNT-10 sample and even lower than the NiGNT-30 sample. With the increase in the NiGNT nanostructures contents, the CTE of M-NiGNT-20 composites decreased to 8.74µm m^−1^ °C^−1^, and this value further dropped to 7.87µm m^−1^ °C^−1^ for M-NiGNT-30. Therefore, with the assistance of the magnetic field, the vertical distribution of NiGNT nanostructures demonstrated an obvious effect in restricting the expansion of the epoxy matrix along the vertical direction, and the increase in fillers could further enhance the restriction.

Table 3 displays the conductive performances reported in the literature of different polymer-based composites with various fillers in comparison to the NiGNT–epoxy composites in this study. In a previous study, the aligned multilayer graphene flakes (GNPs) were uniformly distributed in the epoxy matrix through an external electric field [11]. The electrical resistance and thermal conductivity of obtained composites improved with an increase in fillers amount, and it showed the best conduction performances parallel to alignment direction, compared with the reference samples, which is also close to our results. However, the applied AC field was over 50 V, and the whole facility was complicated. The Fe_3_O_4_-modified graphene samples were aligned in an epoxy resin via a weak magnetic field. As the added amount of fillers was limited, the measured electrical conductivity increased from 10^13^ to 10^9^ Ω m, and the increases in conductivity were attributed to a tunnelling conduction mechanism [5]. Kim reported that Fe_3_O_4_–carbon nanofiber hybrids were aligned to form a chain-like structure in the epoxy resin, but the obtained electrical resistance only showed a minor increase from 10^13^ to 10^8^ Ω m [22]. Although it also demonstrated anisotropic electrical properties, the authors ascribed the relatively high electrical resistance to the oxygen concentration present in the CNFs, resulting in the high percolation threshold in the composites. The enhancement effects of these fillers were similar to the results of this study but all lower than ours. However, as an insulation matrix, all epoxy-based composites did not show impressive enhancements. Most markedly, Ag wires generated by Ag particles in an epoxy matrix with the help of an external electrical field showed a significant improvement in electrical conductivity of the composites, from 10^8^ to 10^2^ Ω m in the alignment direction [36]. However, the high expense of Ag as fillers restricts the further application of this approach. Thus, the reinforcements of aligned NiGNT in epoxy composites can be regarded as a potential candidate to further improve the polymer composites applied in fields such as aerospace, aviation, and automobile manufacturing.

**Figure 11:**
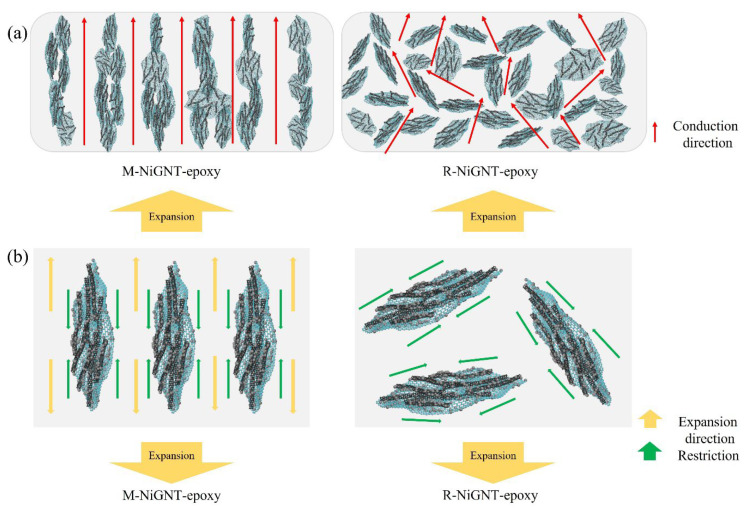
The schematic of conduction (**a**) and expansion (**b**) of the as-prepared pellets.

In summary, in this study, well-dispersed graphene-layer-like NiGNT nanostructures were successfully prepared and aligned in an epoxy matrix with the assistance of a magnetic field. Combined with the back-scattering SEM image shown in Figure 5, it can be concluded that 3D-NiGNT nanostructures were well-dispersed and could be aligned and distributed with the assistance of a magnetic field. The schematic in Figure 11a shows that NiGNT–epoxy composites with magnetic field assistance had 3D bridges in the Z direction of the composite sample, in comparison to the reference composites which involved randomly distributed NiGNT fillers. The enhanced thermal and electrical conduction performances can be ascribed to 3D networks, which were considered as aligned bridges or paths, providing continuous pathways of heat and electrons but also shortening the distance when compared with reference samples. It is interesting to note that the enhancements of samples’ electrical conductivities, both in this study and in previous reports in the literature, were much higher than the enhancements of thermal conductivities. Additionally, this can be ascribed to different conduction theories; for instance, heat is always carried by acoustic phonons in polymers, while electricity is always transferred by electrons [38]. Meanwhile, the improvement in CTE performance can be also ascribed to this aligned 3D network, as the dimension change in the Z direction in the M-NiGNT samples displayed a minor increase in comparison to the reference samples. TEM results showed that NiGNT nanostructures had a good interface with the epoxy matrix (Figure 7), and such aligned distribution of NiGNT nanostructures could, therefore, prevent the expansion of the composite alongside cross-plane direction (Figure 11b), while the random distribution of NiGNT fillers could not restrict this expansion effectively. Thus, it can be concluded that the M-NiGNT composites restricted the thermal expansion of the epoxy matrix, and increasing the number of fillers resulted in better performance.

## 4. Conclusions

In summary, uniform Ni-nanoparticle-decorated GNT (NiGNT) nanostructures were successfully prepared through the MLM process with a subsequent reduction method. The extensive characterisation confirmed the well-dispersed structure and uniform decoration of Ni nanoparticles on the GNTs. With the assistance of a magnetic field, NiGNT nanostructures were uniformly distributed in the epoxy matrix, and the SEM results demonstrated that NiGNT fillers were distributed alongside the direction of the magnetic field. Such a 3D alignment network effectively connected the cross-plane direction of the as-prepared NiGNT–epoxy composites. Due to this 3D alignment network, the NiGNT–epoxy composites showed enhanced thermal conductivity (around 2.67 times that of pure epoxy) and lower electrical resistivity (from around 10^10^ Ω m of pure epoxy to 10^5^ Ω m of NiGNT–epoxy composites). Meanwhile, the CTE of the M-NiGNT composite also improved, and the aligned NiGNT fillers could effectively prevent the expansion of the epoxy matrix alongside the Z direction when compared with the random distribution ones. Therefore, the aligned NiGNT nanostructures in epoxy matrix with enhanced performances inspire the fabrication of high-performance epoxy-based materials, especially where anisotropic properties are desirable, and make them potential candidates to be applied in fields such as aerospace, aviation, and automobile manufacturing.

## Figures and Tables

**Table 1:** Properties of CNTs and GOs.

Sample Name	Size	Metal Content	Oxygen Content
CNTs	6–13 nm in outer diameter × 2.5–20 μm in length	<3.0%	~
GOs	5 μm in lateral dimension × 1 nm in depth	<0.1%	>30%

**Table 2:** Properties of epoxy resin.

Name	Viscosity (mPa·s) at 25 °C	Density (g cm^−3^) at 25 °C	Hardness (Shore D)
Part A	400 ± 100	1.10	
Part B	350	1.04	
Mixture (1:1)	500–1000	1.08	80

**Table 3:** Comparison of conduction performances between NiGNT–epoxy composites and results of previous studies.

Sample	Percentage (Vol.%)	Electrical Resistance (Ω m)	Thermal Conductivity (W m^−1^ K^−1^)	Ref
Fe_3_O_4_–GNP	0.20	10^9^ (aligned)	-	[5]
		10^11^ (Non-aligned)	-	
GNP–epoxy	1.60	10^5^ (aligned)	0.45 (aligned)	[11]
		10^6^ (Non-aligned)	0.40 (Non-aligned)	
Fe_3_O_4_–CF	0.13	10^8^ (aligned)	-	[22]
		10^10^ (Non-aligned)	-	
MWCNT–NR	4.00	-	0.39 (aligned)	[37]
		-	0.35 (Non-aligned)	
Ag–wire	0.40	10^2^ (aligned)	-	[36]
		10^8^ (Non-aligned)	-	
NiGNT	2.50	10^5^ (aligned)	0.46 (aligned)	This work
		10^6^ (Non-aligned)	0.36 (Non-aligned)	

## Data Availability

The data presented in this study are available on request from the corresponding author.
